# Pre-natal and post-natal anxiety in relation to pre-pregnancy obesity: A cohort study on Iranian pregnant women

**DOI:** 10.22088/cjim.11.3.250

**Published:** 2020-05

**Authors:** Azizeh Farshbaf-Khalili, Maedeh Alizadeh, Sakineh Hajebrahimi, Alireza Ostadrahimi, Jamileh Malakouti, Hanieh Salehi-Pourmehr

**Affiliations:** 1 Physical Medicine and Rehabilitation Research Center, Aging Research Institute, Tabriz University of Medical Sciences Tabriz, IR Iran.; 2 Maragheh University of Medical Sciences, Maragheh, Iran.; 3 Research Center for Evidence-Based Medicine, Iranian EBM Centre: A Joanna Briggs Institute (JBI) Center of Excellence, Tabriz University of Medical Sciences, Tabriz, Iran.; 4 Department of Urology, Faculty of Medicine, Tabriz University of Medical Sciences, Tabriz, IR Iran.; 5 Nutrition Research Center, Tabriz University of Medical Sciences, Tabriz, IR Iran.; 6 Department of Midwifery, Faculty of Nursing and Midwifery, Tabriz University of Medical Sciences, Tabriz, IR Iran.

**Keywords:** Body mass index (BMI), Pregnancy, Obesity, Anxiety, Postpartum.

## Abstract

**Background:**

To determine the association between pre-conception obesity and screening results of pre-natal and post-natal anxiety in women that referred to the health centers of Tabriz, Iran.

**Methods:**

62 obese (class 2-3) and 245 normal-weight women were enrolled in the first trimester of pregnancy through the cohort study and followed-up 1 year after childbirth from December 2012 to January 2016. The Beck anxiety inventory scale (BAI-II) was completed in five time points: the first, second, third trimester of pregnancy, 6–8 weeks and 12 months after childbirth. Chi-square, Fisher’s exact tests, Independent *t*-test, Mann-Whitney, and multivariate logistic regression adjusted for confounders were used for data analysis. Statistically significant was considered as p<0.05.

**Results:**

The rate of moderate to severe anxiety in 1^st^, 2^nd^, 3^rd^ trimesters of gestation, 6–8 weeks and 12 months after birth was 8.6%, 10%, 12.6%, 7.8%, 6.5% in normal weight women versus 18%, 17.9%, 19.2%, 12.5%, 19.4% in obese class II women, respectively. The odds of anxiety in the first trimester of pregnancy for class 2–3 obesity was 2.72-fold greater than normal weight group [adjusted odds ratio (aOR) 2.72, 95% confidence interval (CI) 1.14–6.47; p=0.023]. This odd was 3.30- fold (aOR 3.30, 95%CI 1.13-9.60; p=0.045) for 1 year after birth.

**Conclusion:**

Obesity remained associated with positive screening for anxiety in the first trimester of pregnancy and one year after birth. Obese women more likely require special medical care during their pregnancy due to its impacts on mood.

Anxiety disorders during pregnancy are of great significance (1). Its pooled prevalence is 4% for Generalized anxiety disorder (GAD), 15% for any anxiety disorders during pregnancy, and 23% when using cut-offs on validated self-report questionnaires (2). In the previous studies, its incidence range varied from 15.6% (3) to 49.3% (4). The complications of anxiety during pregnancy include preterm labor, low birth weight, low Apgar score at birth, small head circumference, aggravation of hyperemesis symptoms, increased probability of postpartum mood disorder, greater likelihood of cigarette, and alcohol consumption, weak infant-mother relationship, and disability in breastfeeding. Severe and persistent maternal anxiety lead to increment of glucocorticoids levels in serum following the induction of neuroendocrine changes. These changes lead to cognitive or behavioral problems during the prenatal and childhood periods (5-8). 

There are some predictors for development of pregnancy anxiety: hereditary and congenital factors; hormonal changes, pregnancy under 25 years of age, marital status, first pregnancy, physical disorders (9), and social support level (10). The other midwifery predictors are gravidity, the history of stillbirth, abortion, and instrumental delivery or previous cesarean. Also, all of these conditions are strong predictors for postpartum depression, too (11).

It has been assumed that body mass index (BMI) is correlated with mental disorders. Although some evidence points to a correlation between obesity and anxiety, this relationship has not been conclusively demonstrated (12). In addition, relevant studies have been limited in sample size and different methodologies. . Obesity has a negative impact on physical health and mental wellbeing (13, 14). Identification of factors that result in the development and/or aggravation of mental disorders in this period of a woman's life are essential for mothers and infants (15). Studies have always demonstrated the psychopathological relationship between eating, depression and anxiety (16, 17). However, its precise etiology is still unknown. The correlation between weight, mood and anxiety is also uncertain (18). The destructive effect of obesity on physical health is obvious. Obesity is a major cause of morbidity and mortality with a higher burden on healthcare systems(19). 

According to several studies, obese women are more prone to mental disorders than obese men (20-24). Although the effect of obesity on physical health during pregnancy has been widely investigated, and the medical significance and specific effects of obesity on pregnancy have been identified (25), the relationship between obesity and mental health of mothers especially anxiety has been largely ignored. There are limited numbers of studies on the relationship between actual BMI and antepartum and postpartum anxiety (26-28) with contradicting results (29), necessitating the conduction of further studies to identify this relationship. Therefore, the present study was conducted to determine the relationship between pre-pregnancy obesity, anxiety during pregnancy and continued anxiety one year after birth, in women visiting healthcare centers in Tabriz. 

## Methods

**Study population: **Population of the current study were pregnant women referred to health centers in Tabriz, Iran. The research sample included 62 obese in class 2–3 obesity (BMI≥ 35 kg/m2) and 245 normal weight pregnant women (BMI 18.5–24.9 kg/m2) with an age range of 18-35 years old. We followed them from the first trimester of pregnancy till one year after delivery from December 2012 to January 2016. 

**Eligibility criteria: **Inclusion criteria comprised age of 18–35 years, being in the first trimester of pregnancy, being literate, willingness to participate, and singleton pregnancy. Exclusion criteria were smoking or drug abuse, psychiatric medications according to the patient’s declaration, previous history of anxiety and stressful life events in the past 6–12 months at the beginning of study, separation from parents before age of 15, history of chronic diseases, infertility or thyroid dysfunction, physical health problems and signs of threatened abortion in this pregnancy. Participants dropped out of the study due to any problems during gestation and after childbirth, led to loss of pregnancy or the child’s death.

**Sample size: **Sample size was calculated according to the study by *Claesson* et al., (26). Considering P1=9% (prevalence of anxiety among obese pregnant), P2=15.6% (prevalence of anxiety among non-obese pregnant), p=80% and α=0.05, one-sided study plan and 1:4 ratio and using STATA, it was determined as 48 in the obese group and 192 in the normal BMI group. We assumed 20% probability of drop-out in the follow-up period. Finally, sample size was considered as 60 in the obese group and 240 in normal weight group.

**Study design: **This is a cohort study on Iranian pregnant women. After approval of proposal and Ethics Committee confirmation, a list of pregnant women who had prenatal care record in health centers of Tabriz (65 centers and sub-centers) was first prepared. Convenience sampling was used for the selection of eligible obese women among the centers with highest number of pregnant women. Then for every obese woman, four eligible women with normal BMI were enrolled at the same center by using simple random sampling. In the beginning of the study, the study objectives were explained as well as informed consent was obtained and voluntariness and confidentiality of the information was emphasized. Then, demographic, midwifery, and Beck anxiety inventory (BAI-II) questionnaire were given to them.

Pre-pregnancy weight (or the first trimester weight in the absence of pre-pregnancy record) was used for BMI calculation. Also, a tape measure mounted on the wall at health care centers was used for height measuring. Pre-conception BMI less than 18.5 was classified as underweight, between 18.5 and 24.9 as normal weight, between 25.0 and 29.9 as overweight, between 30.0 and 34.9 as obese class 1, between 35.0 and 39.9 as obese class 2, and greater than 40.0 as obese class 3, according to WHO classification (30).To control some confounders like maternal age, singleton pregnancy, literacy, and smoking or drug abuse, eligibility criteria was considered as mentioned at above. For controlling the other confounders such as stressful life events and history of previous pregnancy, statistical method adjustment was considered. If at any stage of pregnancy, fetal abnormality that led to abortion and fetal death happened, we withdraw the included case. To control the confounding factors of socioeconomic status, for each obese participant from every health center, four normal BMI pregnant women were recruited from the same center.

**Data collection tools: **Demographic and midwifery, and Beck anxiety inventory (BAI-II) were the used questionnaires for gathering the data. The eligible recruited women completed both questionnaires in five stages: 3 time-points in different trimester of pregnancy, and two time-points following delivery (6-8 weeks, and 1 year after childbirth). The socio-personal part of the demographic questionnaire contained questions about age, spouse age, level of education, job, income, pre-pregnancy and first trimester weight, and height. This part was followed by items in relation to pregnancy in each trimester comprising wanted or unwanted pregnancy. 

Pregnancy complications including gestational hypertension, vomiting, diabetes, as well as issues like as bleeding were the other questions. Items related to the delivery characteristics (type of delivery, gestational age at birth, sex of newborn, satisfaction of sex, birth weight, Apgar score, need for resuscitation, need for hospitalization, postpartum bleeding, etc.) were completed between the 6 and 8 weeks after childbirth. 

The Beck anxiety inventory (BAI-II) has 21-items. Each item score is between 0-3-point based on the severity of anxiety. The following guidelines are commonly used in interpreting the total score: 0-7 (minimal anxiety), 8-15 (mild anxiety), 16-25 (moderate anxiety), and above 26 (severe anxiety). The validity and reliability of BAI-II questionnaire were evaluated in Iran (31). 

In the present study, scores ≤ 15 were considered as minimal and/or mild anxiety and scores ≥ 16 as moderate to severe anxiety. This questionnaire was given to the participants in a peaceful setting to be completed after explaining the project objectives and emphasizing the voluntary nature of participation. To evaluate the validity of demographic and midwifery questionnaires, they were given to 10 academic members and then corrections had been made according to the comments. To determine the reliability of BAI questionnaire (intra-class correlation coefficient, ICC) and internal consistency (Cronbach’s α coefficient), test and re-test were implemented on 30 participants. ICC [confidence interval (CI) 95%] and Cronbach’s α were 0.83(0.74-0.89) and 0.82 respectively.

**Statistical analysis: **To evaluate the normal distribution of continuous variables, Kolmogorov-Smirnov test was used. To examine the frequency distribution, mean and CI 95% of the mean, descriptive statistics were used. To answer the questions of the research, they used independent t-tests and Mann- Whitney, and for categorical variables Chi-square and Fisher’s exact tests were utilized. To adjust the confounders (including recruitment center, age, husband’s job and residential status), univariate and multivariate logistic regression was applied. A p<0.05 was considered as statistically significant. Data were analyzed using SPSS software (SPSS 21, SPSS Inc., Chicago, IL, USA). 

## Results

**Figure 1:**
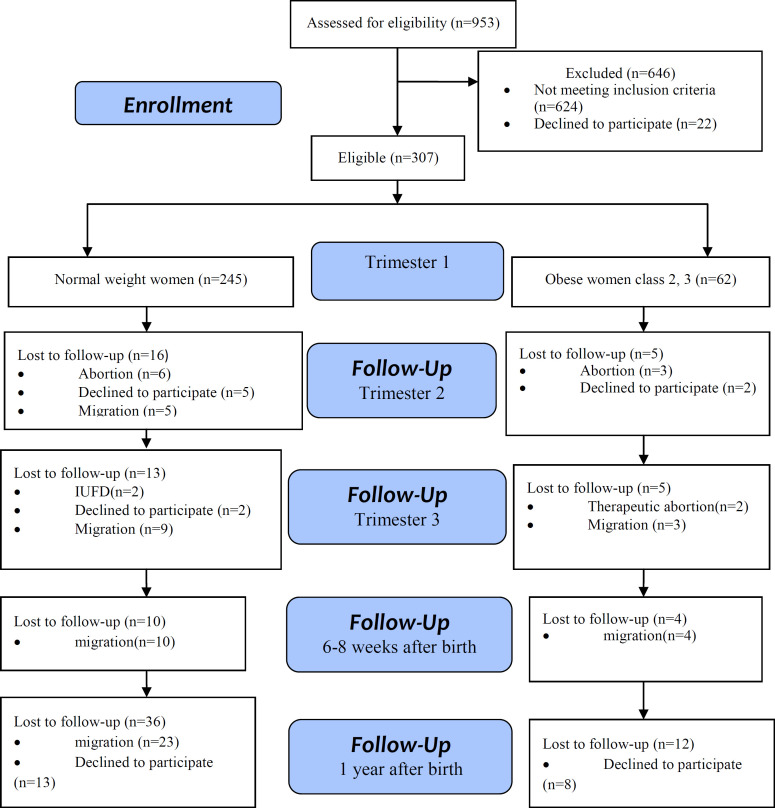
Flow diagram of the progress through the stages of cohort study

Two hundred and forty-five normal BMI and 62 class 2–3 obese subjects among a total of 307 pregnant women selected from 16 health centers in Tabriz completed the demographic and BAI-II questionnaire in the first trimester of pregnancy. Figure 1 illustrates these rates in the 2^nd^ and 3^rd^ trimesters of gestation and after delivery period. Obese women had significantly higher mean age than normal weight women (P=0.001). There were significant differences in husband’s job (P=0.037) and parity (0.007) between two groups (table 1). Within regard to pregnancy outcomes, there were significant differences between two groups according to abortion, cesarean sections, hypertension, GDM (gestational diabetes mellitus), gender of participants’ newborns (p<0.05) (table 2). According to the findings, 8.6% normal weight and 18% of obese developed moderate to severe anxiety during the first trimester. This rate was 10% versus 17.9% in the second trimester, 12.6% versus 19.2% in the third trimester, 7.8% versus 12.5% in 6-8 weeks postpartum and 6.5% versus 19.4% within 1-year after birth respectively (table 3). Obesity was associated with positive screening of anxiety in first trimester [adjusted OR (aOR) 2.72, 95% CI 1.14-6.47], and 1 year after birth (aOR 3.30, 95%CI 1.13–9.60 for) compared with normal weight women adjusted for confounders. Class 2–3 obesity in pregnant women during 1^st^ trimester of gestation increased odds of anxiety 2.72-fold compared to normal weight based on multivariate logistic regression This odd was 3.30-fold in class 2–3 of obesity one year after birth compared to normal weight participants (table 3).

**Table 1:** Baseline demographic and Obstetric data of women stratified by pre-pregnancy BMI category

**p**	**class-2,3 Obese** **n=62**	**Normal** **n=245**	**Total** **N=307**	**General characteristics**
<0.001^a^	28.95(5.02)	25.38(5.58)	26.10(5.65)	Age*mean(SD)
				**Education**
0.23^b^	13(21.0) 14(22.6) 10(16.1) 21(33.9) 4(6.5)	50(20.4) 43(17.6) 35(14.3) 78(31.8) 39(15.9)	63(20.5) 57(18.6) 45(14.7) 99(32.2) 43(14.0)	primary secondary high school diploma academic
				**Husband Education**
0.13^b^	16(25.8) 11(17.7) 11(17.7) 18(29.0) 6(9.7)	34(13.9) 63(25.7) 35(14.3) 77(31.4) 36(14.7)	50(16.3) 74(24.1) 46(15.0) 95(30.9) 42(13.7)	primary secondary high school diploma academic
				**Job **
0.20^c^	58(93.5) 4(6.5) 0(0.0)	225(91.8) 10(4.1) 10(4.1)	283(92.2) 14(4.6) 10(3.3)	Housewife Working at home Working outside
				**Husband Job**
0.037^c^	47(75.8) 13(21.0) 2(3.2)	211(86.1) 33(13.5) 1(0.4)	258(84.0) 46(15.0) 3(1.0)	Private sector employee unemployed
				**Income**
	19(30.6)	78(31.8)	97(31.6)	Income<outcome
0.77^d^	38(61.3)	150(61.2)	188(61.2)	Income=outcome
	5(8.1)	17(6.9)	22(7.2)	Income>outcome
<0.001^ a^	36.35(3.26)	22.30(1.85)	25.03(5.98)	pre-pregnancy BMI*
<0.001^ a^	36.33(3.23)	22.65(2.00)	25.42(5.96)	First trimester BMI*
0.23^a^	1.98(0.96)	1.80(0.78)	1.84(0.82)	Gravida*
0.007^ a^	0.81(0.74)	0.53(0.63)	0.59(0.66)	Para*

*All variables reported by N (%) except age, pre-pregnancy BMI, First trimester BMI, Gravida, Para. a. Independent t-test b. Mann-whitney U c. Fisher’s exact d. Chi-square

**Table2:** Pregnancy outcome, total and stratified by pre-pregnancy BMI category

**p**	**class-2,3 Obese** **n=62**	**Normal** **n=245**	**Total** **N=307**	**Pregnancy outcome**
0.63^a^	0 (0%)	2 (0.8%)	2(0.7%)	IUFD*
0.049^b^	5 (8.1%)	6 (2.4%)	11(3.6%)	Abortion
				**Type of delivery**
0.004^b^	15 (28.8%) 37 (71.2%)	109 (50.9%) 105 (49.1%)	124(40.4%) 142(46.3%)	NVD C/S
0.45^b^	23(41.8%)	109(47.8%)	132(46.6%)	Nausea
0.001^b^	11(20.4%)	10(4.7%)	21(7.9%)	Hypertension
0.001^a^	6(11.5%)	2(0.9%)	8(2.6%)	GDM
0.77^d^	266.81(15.73)	267.51(16.00)	267.37(15.92)	Gestational age at delivery (day)
0.25^c^	3325.58(501.01)	3237.38(473.23)	3254.62(479.10)	Neonate weight Mean (SD)
				**Gender**
0.02^b^	21(40.4%) 31(59.6%)	121(56.3%) 94(43.7%)	142(53.2%) 125(46.8%)	Female male
0.57^a^	3(5.9%)	14(6.6%)	17(5.5%)	Resuscitation of neonate
0.29^b^	8(15.7%)	25(11.7%)	33(12.5%)	Hospitalization of infant in first 8 week
0.48^a^	1(3.3%)	3(1.8%)	4(2.0%)	Hospitalization of infant in first 1 year

allvariabels reported by N (%) except neonate weight and Gestational age at delivery. IUFD: Intra Uterine Fetal Death, GDM: Gestational Diabetes Mellitus a. Fisher’s exact b.Chi- square c. Mann-whitney U d. Independent t-test

**Table 3:** Comparison of the participants with positive screening for anxiety in obese and normal weight group

**Positive Screening (PS)**	**Class 2–3 obese** **(n **=**62)** **N (%)**	**Normal weight** **(n **=**245)** **N (%)**	^a^ **Adjusted** **OR (95%CI)**	**p-value**
PS at first trimester	21(8.6)	11(18.0)	2.72 (1.14-6.47)	0.023
PS at second trimester	23(10.0)	10(17.9)	2.13 (0.89-5.04)	0.086
PS at third trimester	27(12.6)	10(19.2)	1.75 (0.77-3.99)	0.221
PS at 6–8 weeks postpartum	16(7.8)	6(12.5)	2.09 (0.75-5.73)	0.158
PS at 1 year after birth	11(6.5)	7(19.4)	3.30 (1.13-9.60)	0.045

*Cut-off point for positive screening of anxiety based on BAI-II is 15. Normal women were the reference group.

^a^Multivariate logistic regression adjusted for age, parity, residential status, and husband job.            BAI-II = Beck Anxiety Inventory

## Discussion

 This is the first study in Iran, in which two groups of pregnant women with normal weight and class 2, 3 of obesity on the basis of their pre-pregnancy BMIs were screened in terms of anxiety from the first trimester of pregnancy to 1-year after delivery, using the BAI questionnaire. Significant association was revealed between pre-pregnancy obesity and anxiety in 1^st^ trimester of gestation and one year after birth. Obesity as an epidemic has important effects on all individuals specifically reproductive age women (32). Both mothers and infants suffer from short and long-term complications of pre-pregnancy obesity (33-39) including higher rate of abortion, cesarean section, hypertension and gestational diabetes in the current study as well as the previous surveys (40-43). 

In the present study, the obese women were older. There were significant differences with regard to husband’s job and residential status between two groups. These findings are inconsistent with those of *La Coursiere* (43). However, it subscribes to the finding that obese women have better socioeconomic status (44, 45).

The present study showed a correlation between pre-pregnancy BMI and positive results of anxiety screening in the 1^st^ trimester of pregnancy and one year after birth (p<0.05). According to the findings, 18% of class 2, 3 obese women in the first, 17.9% in the second, 19.2% in the third trimester of pregnancy, 12.5% in 6-8 weeks and 19.4% in 1-year after delivery demonstrated positive symptoms for anxiety screening. This ratio in pregnant women with normal weight was 8.6%, 10%, 12.6%, 7.8%, and 6.5%, respectively. *Carter* et al. (2000) did not indicate any association between women’s BMI during pre-pregnancy period and nutritional patterns with increment in anxiety odds during gestation. However, the BMI values were significantly correlated with anxiety symptoms in the 4-month and the 14-month after delivery. Findings illustrated that obese women were more at risk for anxiety in the 4-month postpartum (46). This is nearly consistent with our study results. The study by *Laraia* (2013) (27) showed a relationship between pre-pregnancy obesity, severe obesity, and overweight, (based on maternal self-reports) with enhanced odds for anxiety during gestation. 

The results of a review study indicated that obese and overweight women in comparison with normal weight women are more at risk for anxiety during pregnancy (27). This is consistent with our study results for 1^st^ trimester. The rate of anxiety in obese class I and II women during other trimesters was greater than normal weight pregnant women in present study. Nonetheless, these differences were not statistically significant. 

One of probable cause for inconsistent result for the remaining trimesters of pregnancy is the use of Spielberger’s Trait Anxiety Inventory before 20 weeks’ gestation on 2006 pregnant women (210 morbidly obese) for screening anxiety in *Laraia’s* study. 

The findings showed that obese women with regardless of pregnancy are prone to anxiety. There were some limitations in our study: Since this was a non-interventional study, the relationship showing between pre-pregnancy obesity and anxiety does not necessarily indicate a causal relationship that exists between the mentioned factors. In this study, the correlation between obesity and anxiety was merely studied in women with normal weight or class 2, 3 of obesity rather than all women with different ranges of BMIs. Furthermore, moderate and severe anxiety were incorporated because of low number of pregnant women with severe anxiety. Another limitation was related to relatively high drop-out of participants in the follow-up period, so 307, 286, 254, and only 206 women in 1^st^, 2^nd^, 3^rd^ trimesters of pregnancy, 6–8 weeks postpartum, and1 year postpartum, respectively completed the questionnaires. The dropouts were mainly due to miscarriage, intrauterine fetal death, unwillingness to continue participation in the study and immigration. 

In some cases, follow-up by telephone and follow-up through friends and families were possible for the researcher, while other cases were outside the control of the researcher. Despite these limitations, it was a comprehensive study that screened anxiety during pregnancy and 1 year postpartum among the studied women (with normal BMI and class 2-3 obesity) according to their pre-pregnancy weight. 

In conclusion** t**his study showed an association between obesity and anxiety during the 1^st^ trimester of gestation and one year after birth. Although screening for anxiety is not conducted among the routine antepartum care (according to the study in Washington University, only 20% of gynecologists screened patients for anxiety disorders), it seems necessary for the prevention of anxiety adverse maternal and neonatal consequences. Regardless of the exact mechanism of obesity-anxiety correlation, doing more efforts to conduct practical interventions (47, 48) is required. To conduct further investigations is recommended to identify mood disorders aiming at explaining the correlation between these two states before and during pregnancy, and after delivery to identify and recommend the time of intervention in obese women.

## Compliance with Ethical Standards

**Ethical approval**

This study was approved by local Ethics Committee of Research Deputy of Tabriz University of Medical Sciences, Tabriz, Iran (**code: 91163**). All procedures performed in the studies involving human participants were in accordance with the ethical standards of the institution and/or national research committee and the Helsinki Declaration of 1975, as revised in 2000 (available at http://www.wma.net/e/policy/17-c_e.html) and its later amendments or comparable ethical standards.

## Informed consent

 Informed consent was obtained from all the study participants.
